# Supplementary material for: A nanotrap improves survival in severe sepsis by attenuating hyperinflammation
Source: Nat Commun. 2020 Jul 7;11:3384. doi: 10.1038/s41467-020-17153-0 (PMC7341815; doi:10.1038/s41467-020-17153-0)
Supplement: Supplementary file 1 — Supplementory information [file 41467_2020_17153_MOESM1_ESM.pdf]

Supporting Information (SI)

A Novel Nanotrap Improves Survival in Severe Sepsis by Attenuating Hyperinflammation

Changying Shi[1†], Xiaojing Wang[1†], Lili Wang[1,†], Qinghe Meng[2], Dandan Guo[1], Li Chen[3], Matthew Dai[1], Guirong Wang [2,4], Robert Cooney[2,4], Juntao Luo[1, 2, 4,5*]

[1]Department of Pharmacology, State University of New York Upstate Medical University, Syracuse, NY 13210, USA;

[2] Department of Surgery, State University of New York Upstate Medical University, Syracuse, NY 13210, USA;

[3] Department of Pathology, Baylor Scott and White Medical Center, Temple, TX, 76508, USA;

[4] Sepsis Interdisciplinary Research Center, State University of New York Upstate Medical University, Syracuse, NY 13210, USA;

[5] Upstate Cancer Center, State University of New York Upstate Medical University, Syracuse, NY 13210, USA.

[#] These authors contributed equally to this work.

[*]Correspondence and material requests should be addressed to J.L. (email: luoj@upstate.edu)

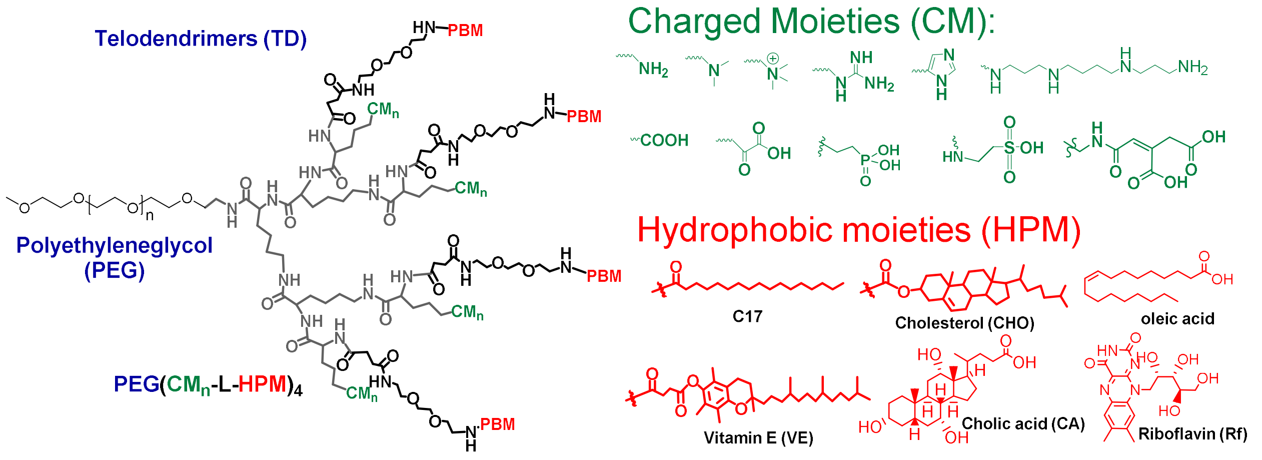

Supplementary Fig. S1. Structures of telodendrimers with varying charge and hydrophobic moieties.

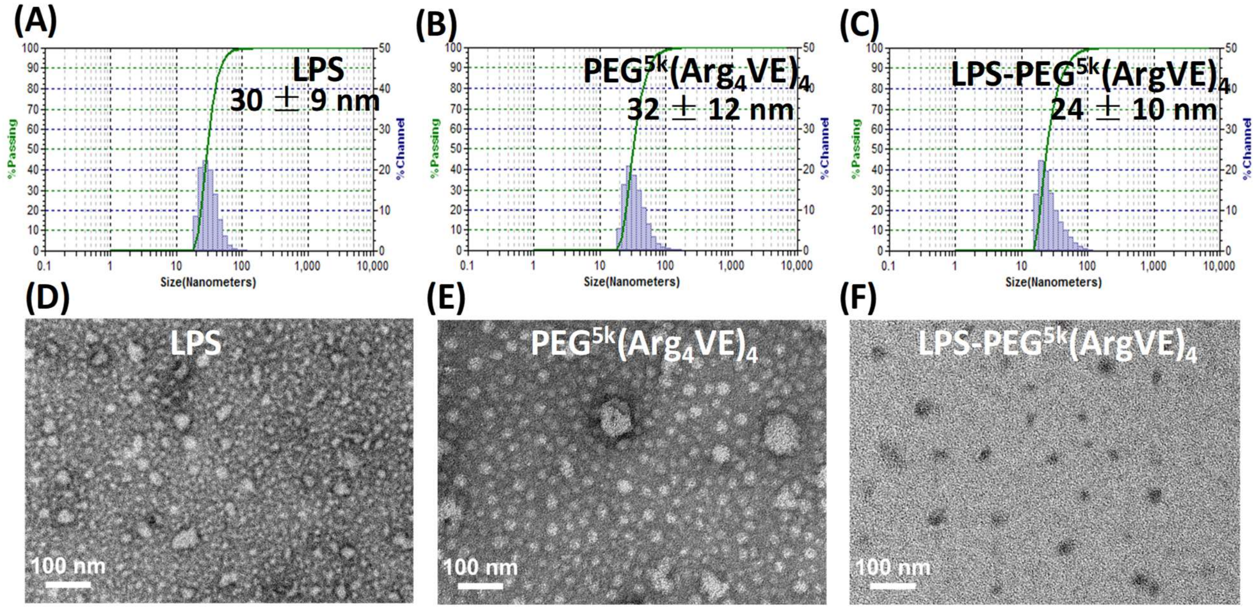

Supplementary Fig. S2. Particle size measurements for LPS trapped in telodendrimers. DLS (A-C) and TEM (D-F, repeated twice independently with similar results) characterization of LPS (A and D), telodendrimer $PEG^{5k}(ArgVE)_4$ (B and E) and LPS loaded $PEG^{5k}(ArgVE)_4$ nanotrap (C and F).

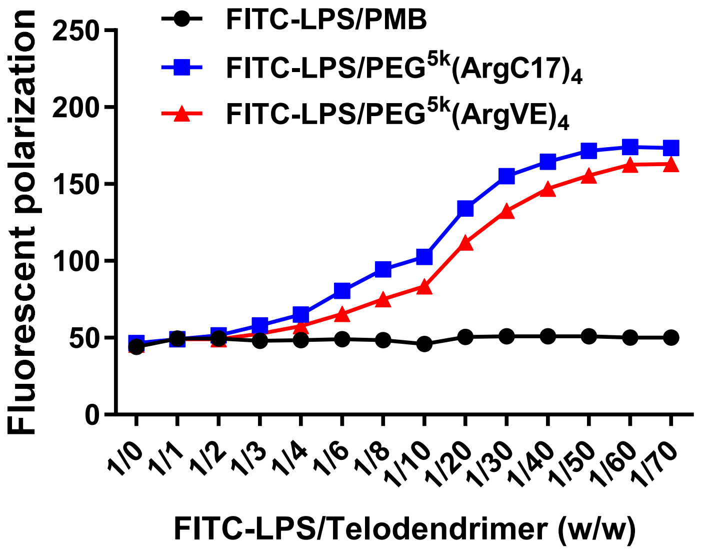

Supplementary Fig. S3. Fluorescent polarization monitoring LPS complexation with TDs or PMB. FITC-LPS (10 µg/mL, < CMC of LPS) form nanocomplex of PMB, $PEG^{5k}(ArgC17)_4$, and $PEG^{5k}(ArgVE)_4$ in PBS monitored by the increased fluorescent polarization (duplicated, mean ± SD).

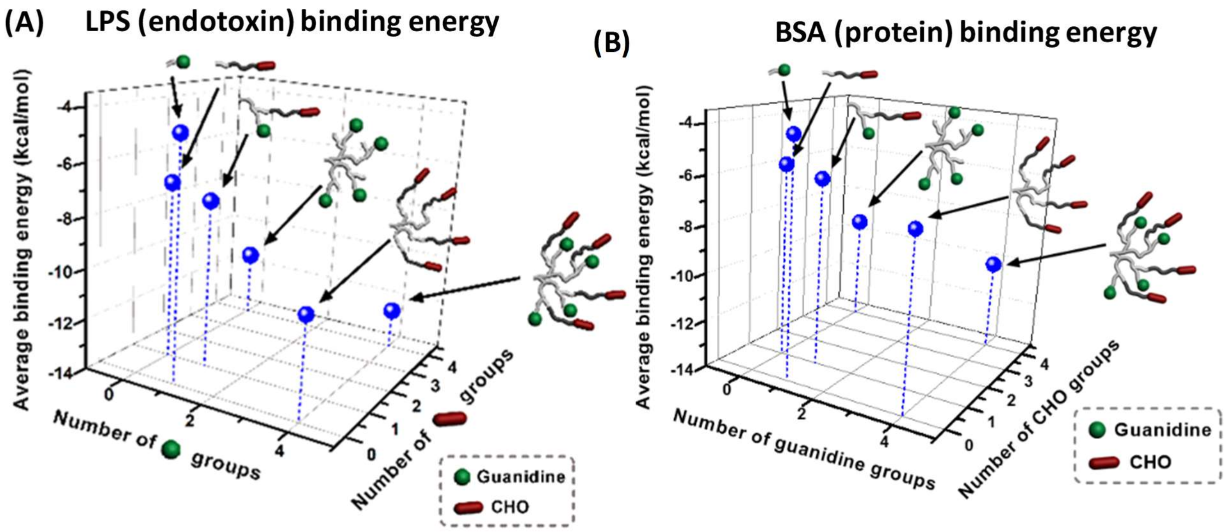

Supplementary Fig. S4. Molecular interactions between LPS-TD and BSA-TD by molecular docking. The average docking energy (an average of 100 docking runs) of different subunits of TD with LPS (A) and BSA (B). The method was detailed in reference [1].

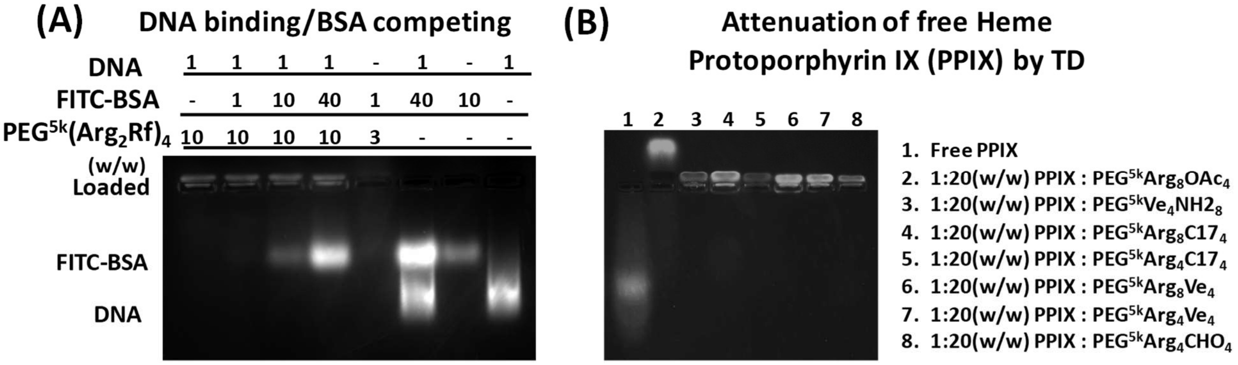

Supplementary Fig. S5. Attenuation of DMAPs/PAMPs molecules. (A) Agarose gel electrophoresis profiles of DNA (from fish sperm) and BSA loading by telodendrimer ($PEG^{5k}(Arg_2Rf)_4$). (B) Agarose gel electrophoresis profiles showing the attenuation of protoporphyrin IX (PPIX) by a series of telodendrimers. (Experiments were repeated more than three times with the similar results.)

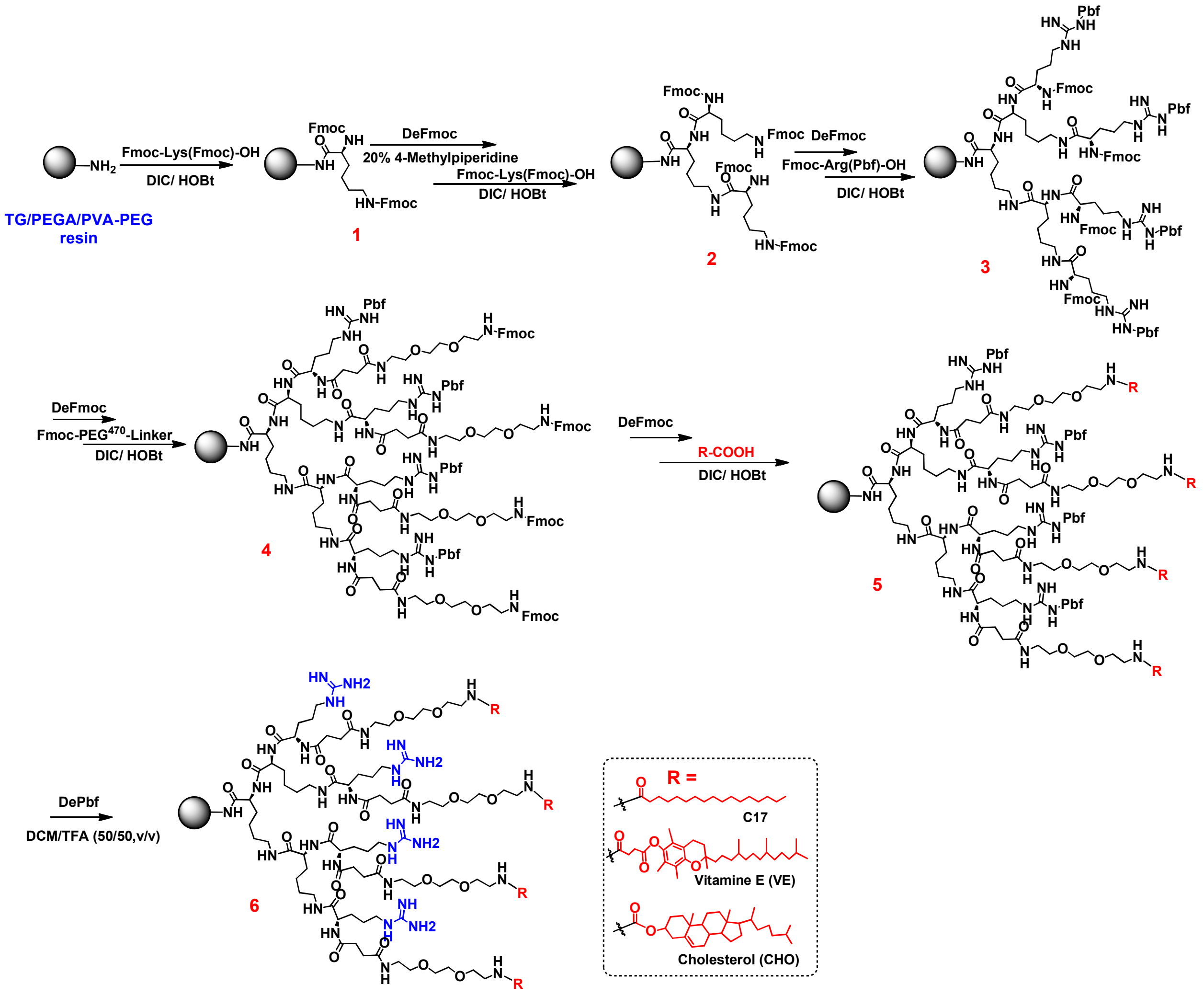

Supplementary Fig. S6. Synthetic scheme for solid phase synthesis of telodendrimers.

(A)

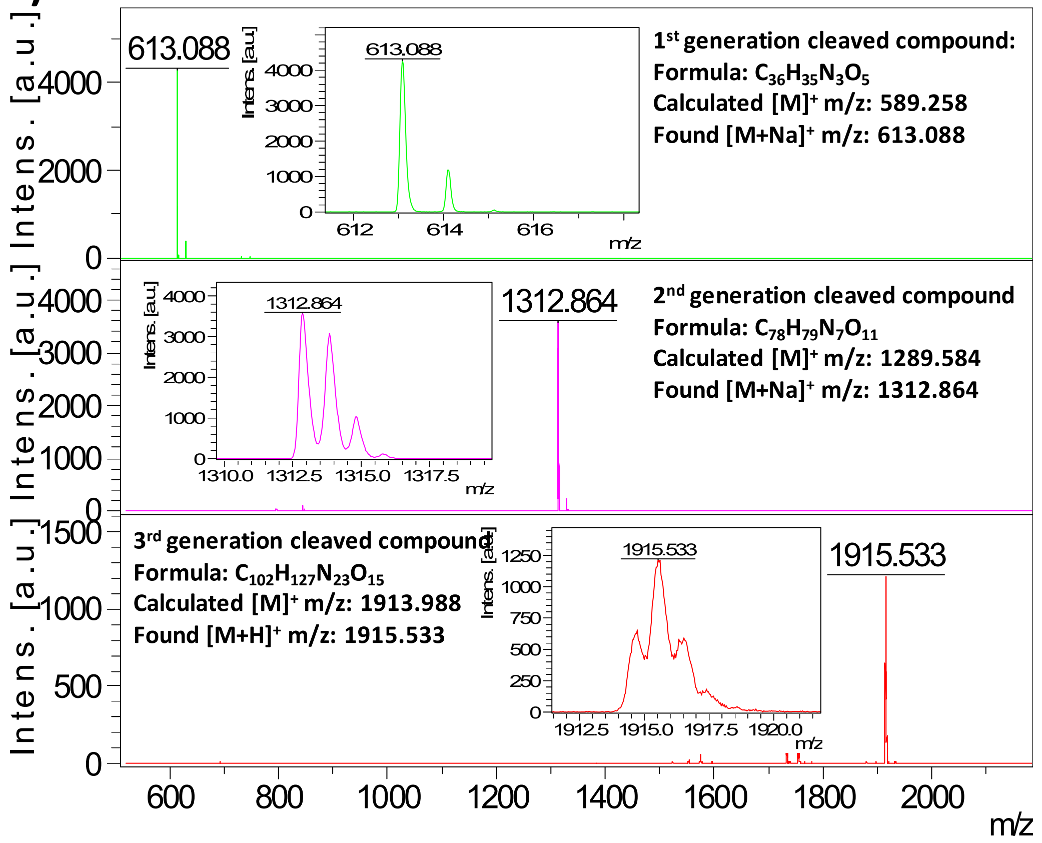

613.088
1st generation cleaved compound:
Formula: $C_{36}H_{35}N_3O_5$
Calculated $[M]^+$ m/z: 589.258
Found $[M+Na]^+$ m/z: 613.088
1312.864
2nd generation cleaved compound
Formula: $C_{78}H_{79}N_7O_{11}$
Calculated $[M]^+$ m/z: 1289.584
Found $[M+Na]^+$ m/z: 1312.864
3rd generation cleaved compound
Formula: $C_{102}H_{127}N_{23}O_{15}$
Calculated $[M]^+$ m/z: 1913.988
Found $[M+H]^+$ m/z: 1915.533
1915.533
Intens. [a.u.]
m/z

(B)

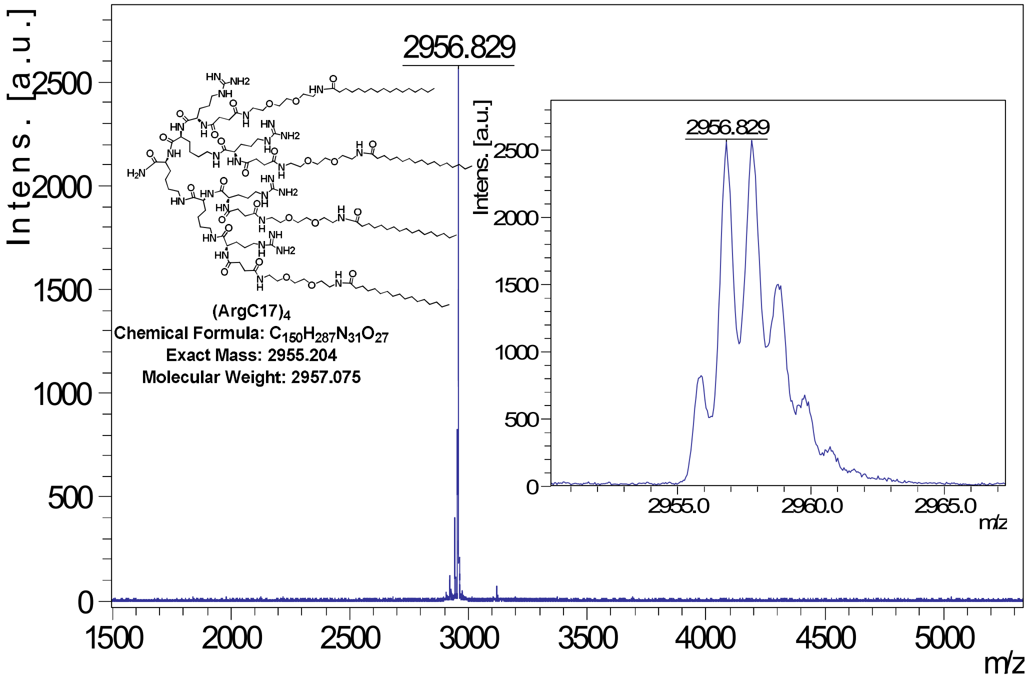

2956.829
(ArgC17)4
Chemical Formula: $C_{150}H_{287}N_{31}O_{27}$
Exact Mass: 2955.204
Molecular Weight: 2957.075
Intens. [a.u.]
m/z

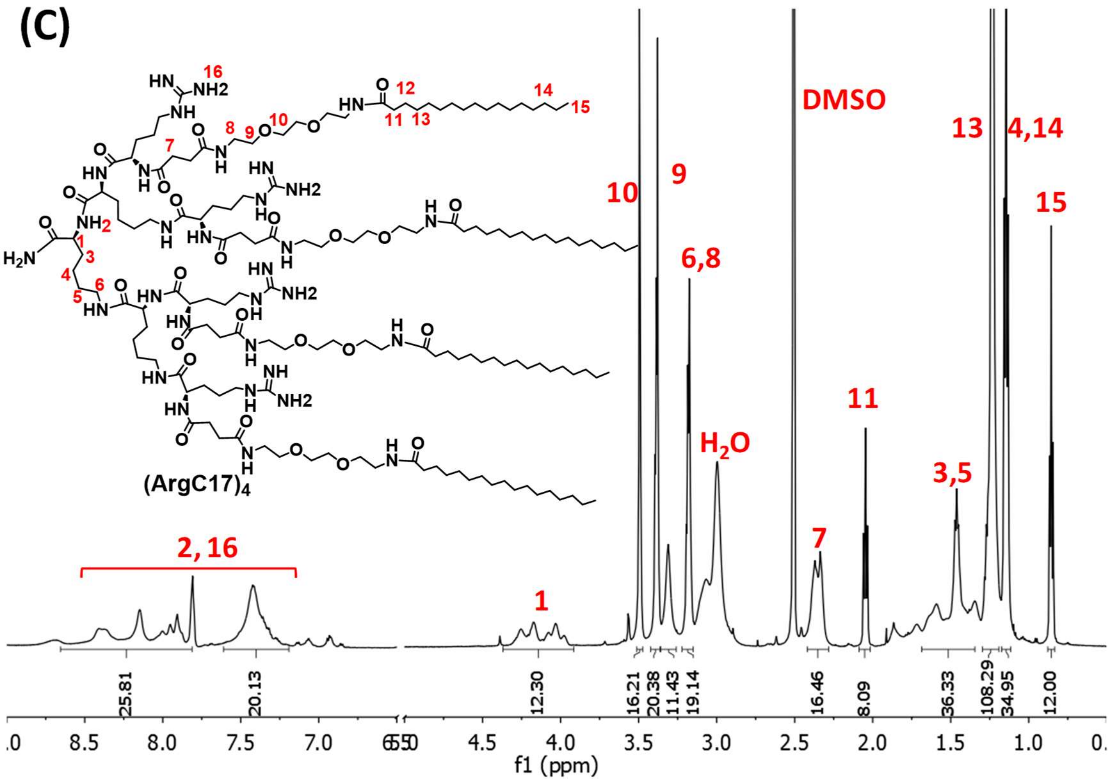

Supplementary Fig. S7. Structural characterizations of TD intermediates synthesized on Rink resin. (A) Stacked MALDI-TOF spectra of intermediates at each dendritic generation. (B) MADLI-TOF spectrum of $(ArgC17)_4$ cleaved from Rink resin. (D) ^{1}H NMR spectrum of $(ArgC17)_4$, recorded in DMSO-d_6.

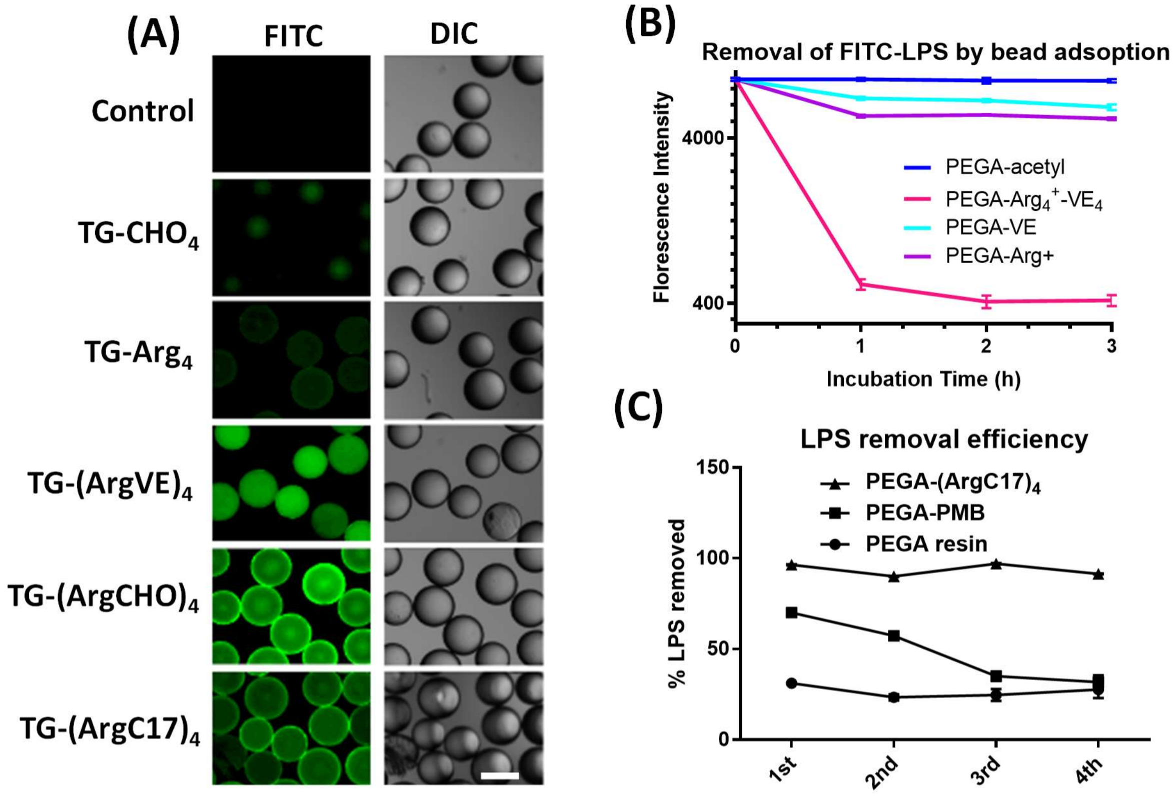

Supplementary Fig. S8. LPS binding and removal efficiency by TD NT resins. (A) Fluorescent microscopy images of FITC-LPS adsorption on PEGylated-polystyrene resin Tentagel (TG) modified with different functionalities after 5 min incubation: the combination of charge and hydrophobic moieties is essential for effective LPS adsorption (Scale bar: 100 μm) Experiments were repeated indecently for more than three times with the similar results.). (B) FITC-LPS removal efficiency of PEGA resins modified with different functionalities (n=3, mean +/- SD). (C) The LPS removal efficiency (2 h incubation in PBS) of PEGA-$(ArgC17)_4$ resin after several cycles of regeneration using 0.2 M NaOH in ethanol.

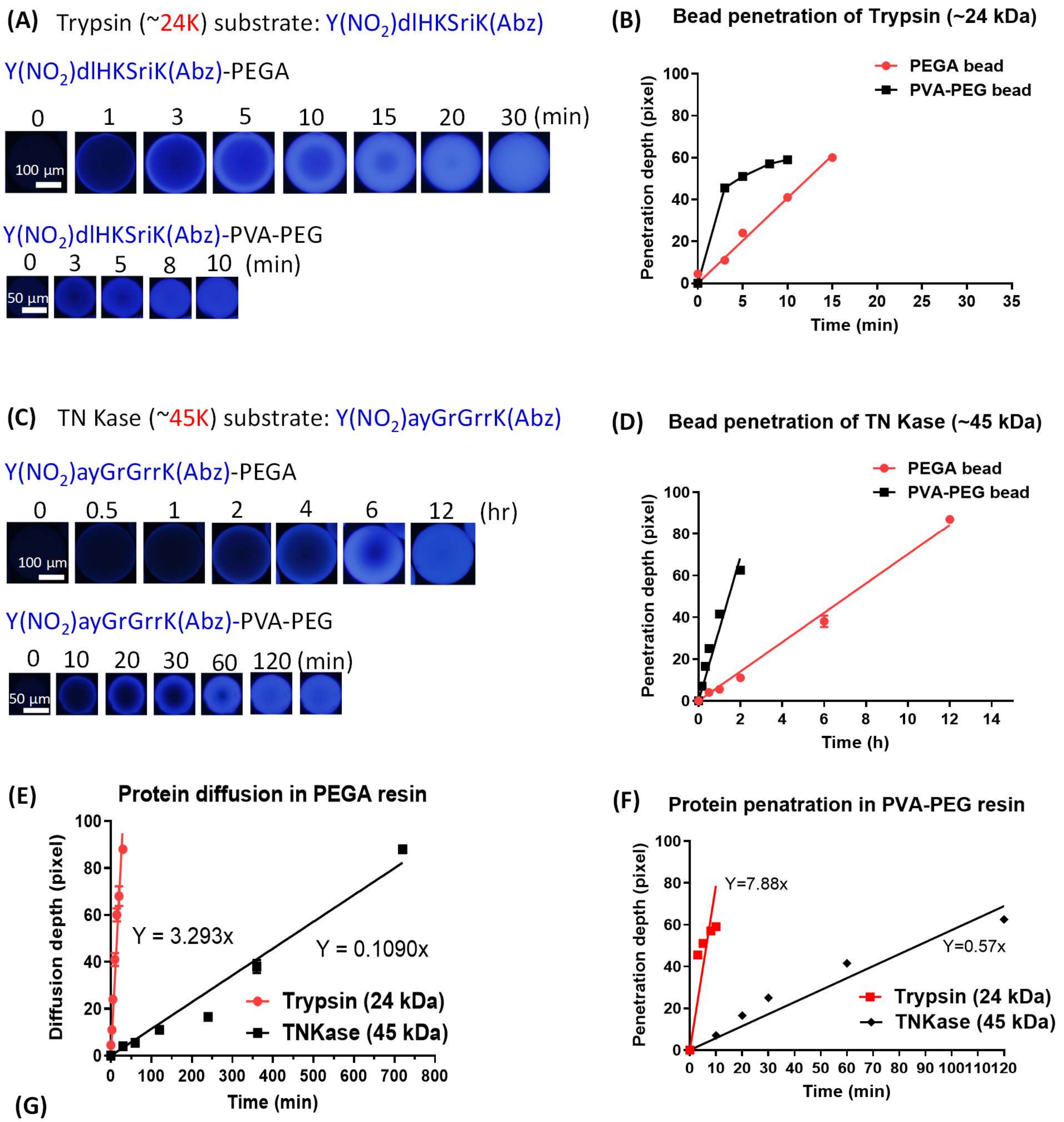

(G)

Diffusion coefficient	PVA-PEG	PEGA	Resin selectivity (PVA-PEG/PEGA)
Trypsin (24 kDa)	7.88 (pix/min)	3.29 (pix/min)	2.40
TNKase (45 kDa)	0.57 (pix/min)	0.109 (pix/min)	5.23
Protein Selectivity	13.8	30.2	0.46

Supplementary Fig. S9. Diffusion kinetics of proteins into PEGA and PVA-PEG resins. Trypsin (24 kDa) and TNKase (Tenecteplase) (45 kDa) were used as model proteins. (A) Confocal

fluorescent images of Trypsin penetration into PEGA and PVA-PEG resin. (B) Kinetic penetration depth vs. incubation time of Trypsin. (C) Confocal fluorescent images of TNKase penetration into PEGA and PVA-PEG resin and (D) Kinetic penetration depth vs. incubation time of TNKase. Kinetic diffusion rates of trypsin and TNKase in (E) PEGA and (F) PVA-PEGA resin, respectively. (G) The diffusion co-efficient and relative selectivity of trypsin and TNKase in PVA-PEG resin and PEGA resin: It revealed that PVA-PEG resin has large pore size than PEGA resin with faster diffusion coefficient for both proteins. However, PVA-PEG resin has a lower selectivity towards smaller protein, i.e. less exclusive for large protein, which will lead to more binding competition by the abundant serum proteins. (Bead incubation and imaging were repeated twice independently with the similar results)

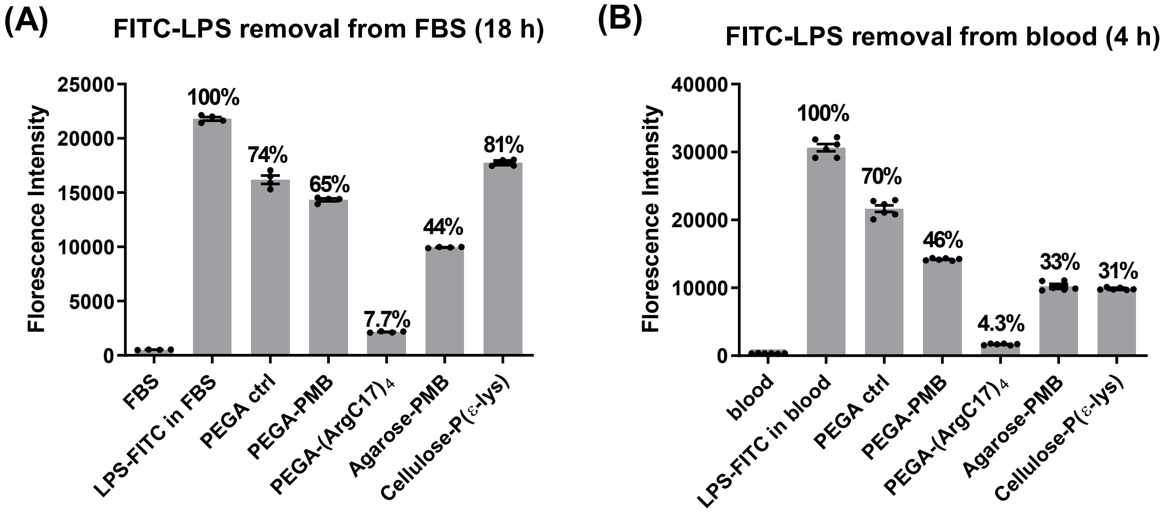

Supplementary Fig. S10. LPS removal efficiency after longer time incubation. FITC-LPS (12.5 μg/mL) was incubated with nanotrap hydrogel resins in comparison with other sorbent resins in FBS (A) or whole blood (B) after 18 h and 4 h incubation, respectively (n=4, mean ± SEM).

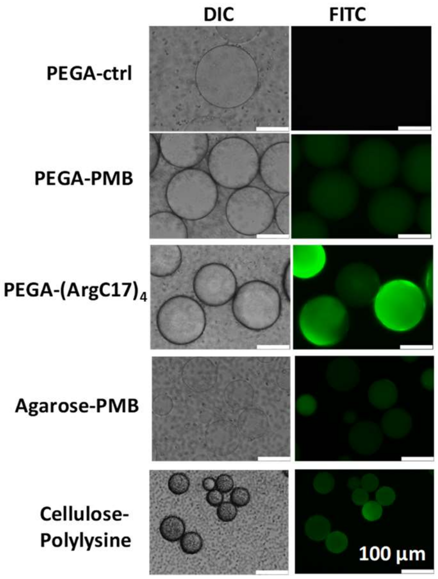

Supplementary Fig. S11. The comparison of LPS adsorption on different resins. Fluorescent microscopy images showed that adsorption of FITC-LPS in TD NT resin was the most efficient than the control resins and commercial LPS-binding resins. Bead incubation and imaging were repeated twice independently with the similar results.

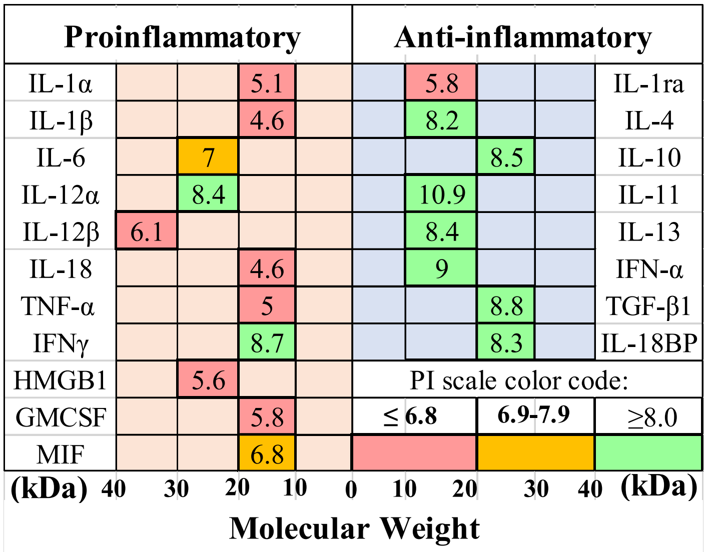

Supplementary Fig. S12. Molecular weights and isoelectric points (PIs) of key cytokines in mouse. There are significant charge disparity in proinflammatory cytokines (negative charge) and anti-inflammatory cytokines (positive charge).

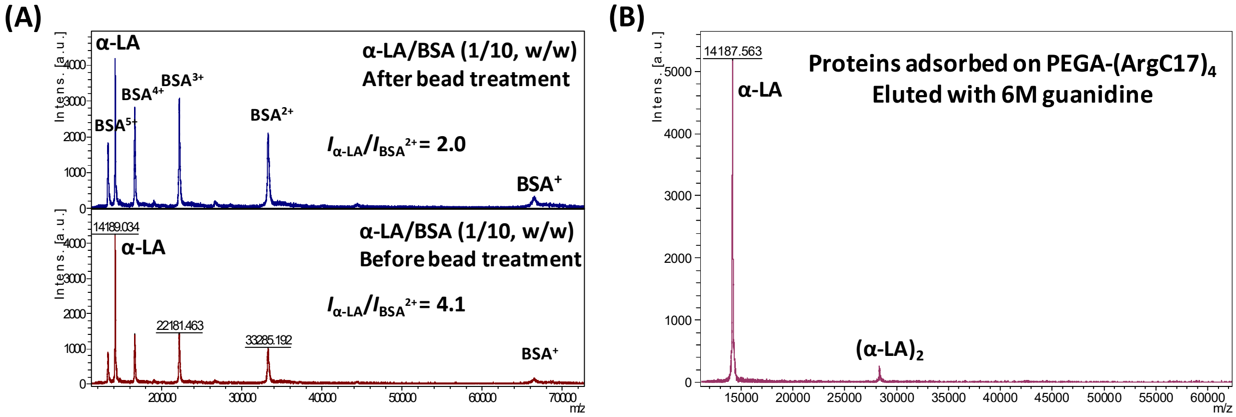

Supplementary Fig. S13. Selective protein adsorption and desorption determined by MALDI-TOF MS. (A) MALDI-TOF MS analysis of the protein mixture solution of α-LA (0.5 mg/mL) and BSA (5 mg/mL) before and after incubation with PEGA-(ArgC17)$_4$ bead at bead/solution ratio of 1:10 (v/v) overnight: Significant reduction of α-LA by ~50% was observed relative to BSA, leading to the saturation of resin with protein and the capacity was calculated to be 13 μg α-LA per mg resin. (B) The MALDI-TOF MS spectrum of proteins eluted from PEGA-(ArgC17)$_4$ resin by 6 M guanidine treatment: only α-LA was detected without observable BSA signals.

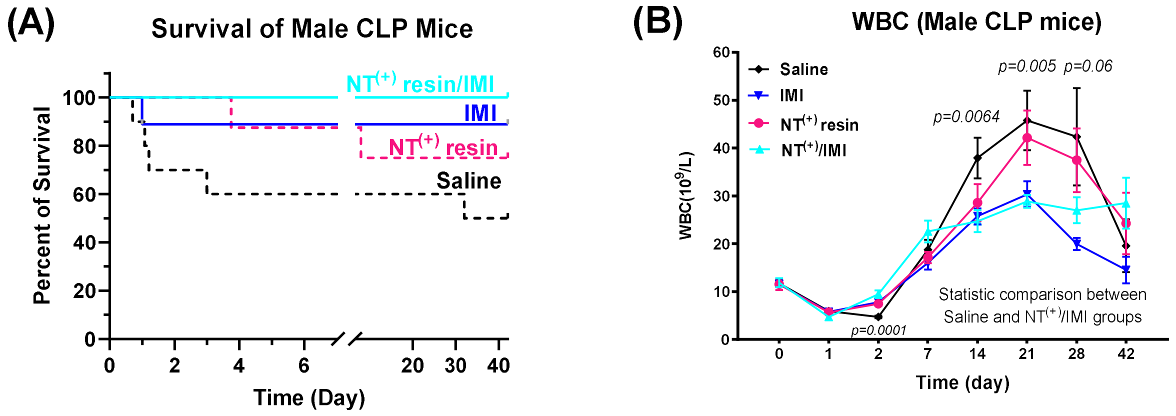

Supplementary Fig. S14 Survival of CLP male mice after treatments. (A) Survival of septic male BLAB/c mice (n=10, 2 month age, male) induced by CLP treated with control saline, $NT^{(+)}$ resin, antibiotics imipenem/cilastatin (IMI, 50 mg/kg, 1:1 by weight) and the combination of $NT^{(+)}$ with antibiotics 3 h post-CLP. (B) Blood cell count analysis revealed the dynamic changes of the white blood cells (WBC) in CLP mice with different treatments over time: mice treated with $NT^{(+)}$/IMI exhibited the most stable hemostasis (n=10 and reduced over time as shown in Fig. S14A, mean +/- SEM). (Statistical significance was measured by unpaired one-sided student's test: * $p<0.05$, ** $p<0.01$, *** $p<0.001$).

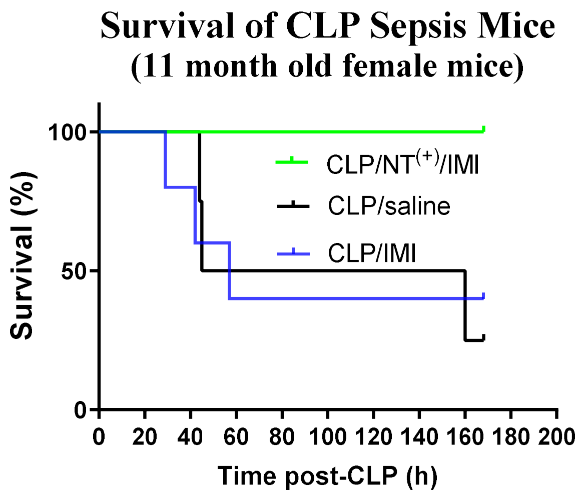

Supplementary Fig. S15. Sepsis treatment in aged mice. The survival of CLP mice (n=5, 11 month old, female) treated with saline or antibiotics imipenem/cilastatin (IMI, 50 mg/kg) with or without positively charged $NT^{(+)}$ PEGA-$(ArgC17)_4$ resin 3 h post-CLP. The survived animals were sacrificed after 7-days observation.

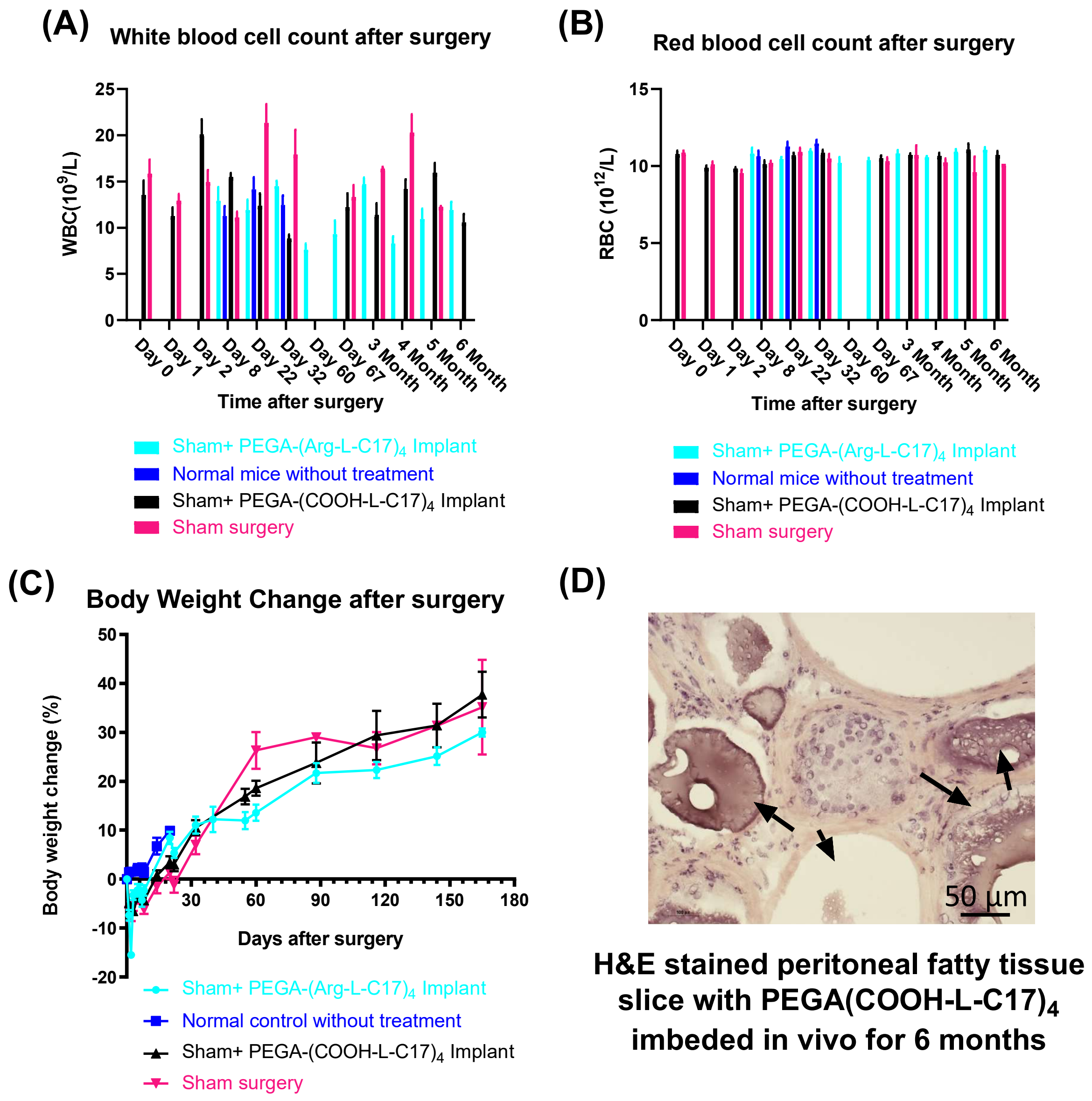

Supplementary Fig. S16. Biocompatibility of TD NT resins after *in vivo* implantation. The white blood cell counts (A), red blood cell counts (B) and body weight changes (C) for mice with intraperitoneal implantation of either positive charged PEGA-(Arg-L-C17)$_4$ or negatively charged PEGA-(COOH-L-C17)$_4$ resins in comparison to the sham mice and untreated mice: no significant variations between groups for all three parameters over six months' observation. (D) The histology studies negative NT resin PEGA-(COOH-L-C17)$_4$ within tissue after six months of in vivo implantation: no active inflammation was observed around resins and partial degradation of resin

was observed (arrowed). (n=5, mean +/- SEM, Multiple tissue slices (n=5) were stained and imaged with the similar results.

References

1. Wang, X., Shi, C., Zhang, L., Bodman, A., Guo, D., Wang, L., Hall, W.A., Wilkens, S. & Luo, J. Affinity-controlled protein encapsulation into sub-30 nm telodendrimer nanocarriers by multivalent and synergistic interactions. *Biomaterials* **101**, 258-271 (2016).
