# Supplementary material for: A nanotrap improves survival in severe sepsis by attenuating hyperinflammation
Source: Nat Commun. 2020 Jul 7;11:3384. doi: 10.1038/s41467-020-17153-0 (PMC7341815; doi:10.1038/s41467-020-17153-0)
Supplement: Supplementary file 3 — Reporting Summary [file 41467_2020_17153_MOESM3_ESM.pdf]

nature research

Corresponding author(s): Juntao Luo

Reporting Summary

Nature Research wishes to improve the reproducibility of the work that we publish. This form provides structure for consistency and transparency in reporting. For further information on Nature Research policies, see Authors & Referees and the Editorial Policy Checklist.

Statistical parameters

When statistical analyses are reported, confirm that the following items are present in the relevant location (e.g. figure legend, table legend, main text, or Methods section).

n/a	Confirmed	
☐	☒	The exact sample size (n) for each experimental group/condition, given as a discrete number and unit of measurement
☐	☒	An indication of whether measurements were taken from distinct samples or whether the same sample was measured repeatedly
☐	☒	The statistical test(s) used AND whether they are one- or two-sided<br>*Only common tests should be described solely by name; describe more complex techniques in the Methods section.*
☒	☐	A description of all covariates tested
☒	☐	A description of any assumptions or corrections, such as tests of normality and adjustment for multiple comparisons
☐	☒	A full description of the statistics including central tendency (e.g. means) or other basic estimates (e.g. regression coefficient) AND variation (e.g. standard deviation) or associated estimates of uncertainty (e.g. confidence intervals)
☐	☒	For null hypothesis testing, the test statistic (e.g. F, t, r) with confidence intervals, effect sizes, degrees of freedom and P value noted<br>*Give P values as exact values whenever suitable.*
☒	☐	For Bayesian analysis, information on the choice of priors and Markov chain Monte Carlo settings
☒	☐	For hierarchical and complex designs, identification of the appropriate level for tests and full reporting of outcomes
☒	☐	Estimates of effect sizes (e.g. Cohen's d, Pearson's r), indicating how they were calculated
☐	☒	Clearly defined error bars<br>*State explicitly what error bars represent (e.g. SD, SE, CI)*

Our web collection on statistics for biologists may be useful.

Software and code

Policy information about availability of computer code

Data collection	no software was used for data collection
Data analysis	All data process and statistical analyses were performed using GraphPad Prism 7.0 (GraphPad Software). Quantification of Western blot was analyzed via Image J software (1.53a)

For manuscripts utilizing custom algorithms or software that are central to the research but not yet described in published literature, software must be made available to editors/reviewers upon request. We strongly encourage code deposition in a community repository (e.g. GitHub). See the Nature Research guidelines for submitting code & software for further information.

Data

Policy information about availability of data

All manuscripts must include a data availability statement. This statement should provide the following information, where applicable:

- Accession codes, unique identifiers, or web links for publicly available datasets
- A list of figures that have associated raw data
- A description of any restrictions on data availability

All data associated with this study are present in the paper or the Supplementary Materials. Correspondence and material requests should be addressed to J.L. The source data underlying Figs. 3A-C, 4B-C, 5B, 5D-F, 6B-D, 7B-C, 8A-I and S3, S10B, S14A-B, S15 and S16A-C are provided as a Source Data file.

Field-specific reporting

Please select the best fit for your research. If you are not sure, read the appropriate sections before making your selection.

☒ Life sciences ☐ Behavioural & social sciences ☐ Ecological, evolutionary & environmental sciences

For a reference copy of the document with all sections, see nature.com/authors/policies/ReportingSummary-flat.pdf

Life sciences study design

All studies must disclose on these points even when the disclosure is negative.

Sample size	sample size (n=10) for animal studies were referenced from literature (Cho, E., Doh, K., Park, J. et al. Zwitterionic chitosan for the systemic treatment of sepsis. Sci Rep 6, 29739 (2016).) with the similar experimental design. Statistical analysis was performed to reveal the significance between groups.
Data exclusions	Animal death by accident due to anesthesia was excluded in the study
Replication	All data were suitably replicated: in vitro adsorption experiments were triplicated with repeated measurements; the analysis of biospecimens were duplicated in ELISA or multiplex immune assay. In vivo sepsis treatment study were repeated in animals for three times in mice with different gender and age populations.
Randomization	Mice from different cages were randomized for control and procedure groups. After CLP surgery, animals were randomized into treatment groups to eliminate the variation from both procedure and individual animal background.
Blinding	Animals were randomized into treatment groups and the animal death rate is the end point for comparison, and there is no bias on judgment, therefore, animal study was not blinded.

Reporting for specific materials, systems and methods

Materials & experimental systems

n/a	Involved in the study	
☒	☐	Unique biological materials
☐	☒	Antibodies
☐	☒	Eukaryotic cell lines
☒	☐	Palaeontology
☐	☒	Animals and other organisms
☒	☐	Human research participants

Methods

n/a	Involved in the study	
☒	☐	ChIP-seq
☒	☐	Flow cytometry
☒	☐	MRI-based neuroimaging

Antibodies

Antibodies used	HMGB-1(Novus Biologicals, NBP2-62767); IL-1β (Invitrogen, BMS6002); IL-6 (Invitrogen, BMS603-2); TNF-α (Invitrogen, BMS607HS); NF-κB (Santa Cruz, sc-8008, 1:200), P-iκB-α (Santa Cruz, sc-8404, 1:200) and β-actin (Santa Cruz, sc-47778, 1:500) HRP-conjugated secondary antibody (Santa Cruz, sc-516102, 1:4,000).
Validation	We used the validated commercial ELISA kits and followed manufacturers guidelines strictly for ELISA assay. Standard curve were collected using the standard proteins provided in the ELISA kit. Antibodies for western blot were validated using positive control with the reference of protein ladders of molecular weight.

Eukaryotic cell lines

Policy information about [cell lines]()

Cell line source(s)	RAW 264.7 (ATCC® TIB-71™)
Authentication	The commercial available cell line was purchased from ATCC, no further authentication was performed in lab after receiving.
Mycoplasma contamination	Cell line RAW 264.7 was tested negative for mycoplasma contamination.

Commonly misidentified lines (See ICLAC register)	No misidentified cell lines were used in this study

Animals and other organisms

Policy information about studies involving animals; ARRIVE guidelines recommended for reporting animal research

Laboratory animals	BALB/c mice, 8-10 weeks or 11 months, both genders from Charles River (USA) were used for mouse experiments.
Wild animals	No wild animals ere used in the study
Field-collected samples	No field collected samples were used in the study
